# Editorial: The role of neuropsychiatry in neurodegenerative disorders

**DOI:** 10.3389/fpsyt.2026.1781963

**Published:** 2026-01-23

**Authors:** Chengyu Sheng, Wang Zheng, Huan Yang, Xi Chen

**Affiliations:** 1 Department of Pharmacology, School of Basic Medical Sciences, Nanjing Medical University, Nanjing, China; 2 National Institute of Allergy and Infectious Diseases, National Institutes of Health, Bethesda, MD, United States; 3 Department of Psychiatry, Second Xiangya Hospital, Central South University, Changsha, China; 4 Institute of Neurology, Sichuan Provincial People’s Hospital, University of Electronic Science and Technology of China, Chengdu, China

**Keywords:** Alzheimer disease, epilepsy, interferon, neurodegenarative disease, neuropsychiatry, Parkinson‘ s disease

Patients with neurodegenerative disorders (NDDs) frequently present with psychiatric symptoms. In some cases, the association is sufficiently robust that these symptoms are proposed as potential biomarkers for evaluating or predicting the development of core cognitive or motor deficits. From a reductionist perspective, both psychiatric and cognitive/motor symptoms have their neurobiological basis, which, in many cases, is actually indistinguishable at the subcellular level. Taking microglia as an example, while neuroinflammation is a common theme, can you tell whether a microglial cell is from a particular kind of neurodegenerative or neuropsychiatric disorder by looking at it or checking its -omics signatures? Thus, it is more convincing that the specific neuronal populations affected, the particular networks disrupted, and the nature of these alterations together determine whether the clinical phenotype manifests as cognitive impairment, motor dysfunction, psychiatric symptoms, or a combination. From such a perspective, more attention should be placed on psychiatric symptoms in NDDs, not only because they bring much pain to patients and their caregivers, but also because they equally imply neurobiological alterations beneath that are possibly shared with the “main” cognitive/motor symptoms, and successful management of the neurobiological alterations is likely to attenuate all those symptoms.

Focused on psychiatric symptoms in NDDs, this Research Topic comprises five original clinical research articles, one review, one mini-review, and one opinion article. Four of the five research articles investigate psychiatric symptoms in Parkinson’s disease (PD). Zhou et al. identified a significant positive linear association between systemic inflammatory biomarkers and anxiety levels, particularly state anxiety, in PD patients. Zhang et al. explored the predictive value of the triglyceride-glucose index—a marker of systemic insulin resistance—for depression risk in PD, and proposed that dysregulated glucose-lipid metabolism and immune-inflammatory pathways serve as a common pathological basis for both PD and depression. Zhu et al. reported that PD patients with multiple concurrent negative psychiatric symptoms, including demoralization, apathy, and depression, exhibit more pronounced cognitive impairment and more severe motor deficits, with cognitive impairment partially mediating the relationship between psychiatric and motor symptoms. Finally, Varlıbaş et al. found a significant positive correlation between depression severity and sexual dysfunction in PD, both of which are critical non-motor symptoms. The fifth clinical study article, by Trieu et al., demonstrated that neuroticism/somatization levels modulate the association between amyloid status and depressive/anxiety symptoms in subjective cognitive decline, suggesting that preclinical Alzheimer’s disease (AD) patients with certain personality traits may be at higher risk for depression.

In the review article, Sirkis et al. comprehensively discussed the role of interferon (IFN) signaling in neurodegeneration and neuropsychiatric disorders. Although primarily recognized as an antiviral pathway, heightened IFN responses are implicated in major neurodegenerative and neuropsychiatric conditions. The authors described how cytoplasmic nucleic acids trigger IFN signaling, thereby driving disease progression through enhanced neuroinflammation. An interesting part is that they also reviewed the modulation of this IFN response by sex and genetic ancestry. In the mini review article, Sone and Kanemoto reviewed the neuropsychiatric aspects of epilepsy. The onset of epilepsy is often associated with preceding or succeeding psychiatric symptoms, providing a good chance to study the neurobiological basis for psychiatric symptoms in a model with objective abnormalities already established. In the opinion article, Rao et al. discussed neurobiological alterations underlying agitation in AD and potential interventions involving melatonin or the CB1 receptor agonist THC.

In conclusion, this Research Topic integrates clinical studies and reviews from basic research perspectives in human and animal models. The Research Topic highlights the pervasive yet frequently overlooked neuropsychiatric symptoms in NDDs, provides in-depth analysis of their underlying neurobiology, and explores potential therapeutic interventions for specific psychiatric conditions in defined contexts. Through iterative, mutually-inspired research efforts on clinical observation, mechanistic investigation, and interventional research, we believe effective management of these neuropsychiatric symptoms—and neurodegeneration itself—will eventually be achieved.

